# Investigation of Thermal Effects of Photocoagulation on Retinal Tissue Using Fine-Motion-Sensitive Dynamic Optical Coherence Tomography

**DOI:** 10.1371/journal.pone.0156761

**Published:** 2016-06-06

**Authors:** Kazuhiro Kurokawa, Shuichi Makita, Yoshiaki Yasuno

**Affiliations:** 1 Computational Optics Group, University of Tsukuba, Tsukuba, Ibaraki, Japan; 2 Computational Optics and Ophthalmology Group, Tsukuba, Ibaraki, Japan; Simon Fraser University, CANADA

## Abstract

To enable an objective evaluation of photocoagulation, we characterize thermal tissue changes induced by laser irradiation with different laser parameters using optical coherence tomography (OCT). Spectral-domain OCT with a newly developed image processing method was used to monitor the thermal changes of *ex vivo* porcine retina. A sequence of OCT B-scans was obtained at the same retinal position simultaneously with the photocoagulation. Cross-sectional tissue displacement maps with respect to an OCT image taken before laser irradiation were computed for images taken before, during, and after laser irradiation, by using a correlation-based custom algorithm. Cross-sectional correlation maps (OCT correlation maps) were also computed from an OCT image taken before laser irradiation as a base-line to visualize alterations of tissue microstructure induced by laser irradiation. By systematically controlling laser power and exposure times, tissue displacements and structural changes of 200 retinal regions of 10 porcine eyes were characterized. Thermal tissue changes were characterized by B-scan images, OCT correlation maps, and tissue displacement maps. Larger tissue deformation was induced with higher laser power and shorter exposure time, while the same total laser energy (10 mJ) was applied. The measured tissue displacements revealed the complicated dynamics of tissue displacements. Three types of dynamics were observed; lateral expansion, lateral constriction, and a type showing more complicated dynamics. The results demonstrated the ability of this OCT-based method to evaluate retinal changes induced by laser irradiation. This evaluation could lead to further understanding of thermal effects, and increasing reproducibility of photocoagulation therapy.

## Introduction

Photocoagulation is a widely used method for treating eye diseases such as diabetic retinopathy [1,2], retinal vein occlusion [3,4], and retinal detachment. Although the treatment is effective and a low burden [5], there is a risk of excessive and irreversible laser damage to the retina, because thermal effects to the retina are not fully known. In particular, photocoagulation with a reduced exposure time increases the risk of overtreatment [6,7], although it reduces pain [8,9] and collateral damage to the neural retina [6,10]. Therefore, monitoring is required to objectively evaluate therapeutic effects on the retina, during and after photocoagulation.

Among monitoring techniques such as the fundus camera, fluorescence angiography, and the infrared camera, optical coherence tomography (OCT) has specific advantages. First, OCT enables non-invasive and non-contact retinal imaging, without any contrast agent. Second, OCT provides depth-resolved high-resolution retinal images, that enable detection of long-term thermal tissue changes induced by photocoagulation, even if the thermal change is ophthalmoscopically invisible [11–15]. Third, OCT can directly monitor the short-term thermal changes of retinal tissues, which induce mechanical displacement during the laser irradiation. This monitoring has been performed with the help of phase-sensitive measurements [16–19], speckle tracking [20,21], and correlation coefficients [22–25]. Therefore, OCT is a promising tool for the further understanding of thermal changes induced by photocoagulation, and may lead to improved reproducibility of photocoagulation therapy, by objectively evaluating possible laser damage to the retina [17,26].

Müller *et al*. used phase-sensitive OCT in addition to standard OCT to investigate tissue changes induced by a coagulation laser, with systematically selected configurations of exposure time, laser power, and spot sizes [17]. Nanoscale axial displacement measurements provide precise dynamic tissue changes during the laser irradiation. Recently, we developed a method to measure in-plane and out-of-plane tissue displacements using correlation coefficients of OCT [25], where in-plane displacement means the displacement within the OCT image plane. Other displacement is denoted as out-of-plane. This method successfully measured tissue displacements and visualized dynamic changes of laser-irradiated retinal tissue. However, in the previous study [25], this method was applied to only a single case, therefore the effects of laser power and exposure time on thermal changes of retinal tissue were not thoroughly examined.

In the present study, we measured tissue displacement and structural changes at 200 retinal coagulation spots of 10 porcine eyes by OCT, by systematically controlling the exposure time and laser power. A custom-designed OCT image processing method, which is an improved version of our previous method [25], was used to analyze mechanical and microstructural alterations of tissue induced by photocoagulation. The dependence of tissue dynamics on laser power and exposure time were also investigated.

## Methods

Spectral-domain OCT (SD-OCT) with a newly developed image processing method was used to monitor thermal changes of *ex vivo* porcine retinas induced by laser irradiation. A sequence of OCT B-scans was taken at the same position of the retina, simultaneously with photocoagulation. Tissue displacements were computed by taking a correlation between a reference OCT B-scan image taken before laser irradiation and target OCT B-scan images taken before, during, and after laser irradiation. By systematically controlling laser power and exposure times, tissue displacements and structural changes at 200 irradiation spots on the retinas of 10 porcine eyes were characterized.

### SD-OCT system

**Fig 1:**
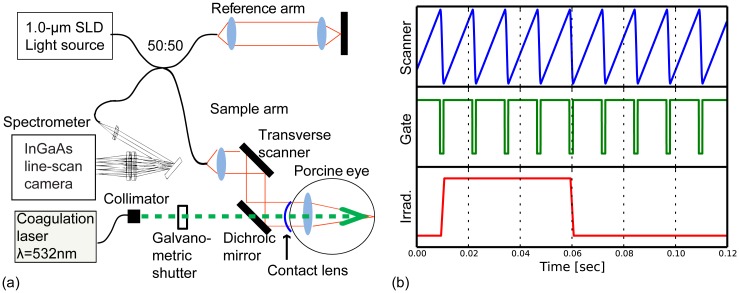
Experimental setup and timing chart. (a) Experimental setup. (b) Timing chart of the galvanometric scanner and image acquisition. Scanner, waveforms to scan the retina; Gate, a gate signal for OCT acquisition; Irrad., the coagulation laser output with an exposure time of 50 ms.

An SD-OCT system, which was custom built by the Computational Optics Group at the University of Tsukuba [24,25], was used in this study. The schematic of the system is shown in Fig 1(a). The system used a superluminescent diode light source (S-1020-B-7; Superlum Ltd., Ireland) with a center wavelength of 1.02 μm and a spectral bandwidth of 100 nm. The interferometer was a fiber Michelson interferometer with a 50/50 fiber coupler. The interference signal was detected by a high-speed spectrometer comprising an InGaAs line sensor (SUI1024LDH2; Sensors Unlimited, Inc., Goodrich, NC, USA) operating at a line rate of 91,911 A-scans/s. The probe beam power was 1.86 mW on a sample, and the sensitivity was measured to be 95 dB.

### Coagulation laser system

The sample arm of the SD-OCT system was equipped with a coagulation laser (532 nm wavelength, GYC-1000; Nidek Co., Ltd., Aichi, Japan) as shown in Fig 1(a). The coagulation laser and the OCT probe beam were combined using a dichroic mirror, and the coagulation laser and the center axis of the OCT probe beam were coaxially aligned so that the OCT B-scan contains the center of coagulation laser. The correct alignment was further confirmed by taking 3-D OCT volumes before and after the laser irradiation and observing the deformation. The galvanometric scanner installed between the laser output and the dichroic mirror was used as a high-speed shutter to synchronize image acquisition with the laser irradiation, so that laser irradiation during the rise/fall time was delivered only during the dead time of the OCT acquisition. The timing chart of the galvanometric scanner and the image acquisition are shown in Fig 1(b). Theoretical beam diameter on the retina is 83 μm, and intensity profile is assumed to be tophat.

### Processing

**Fig 2:**
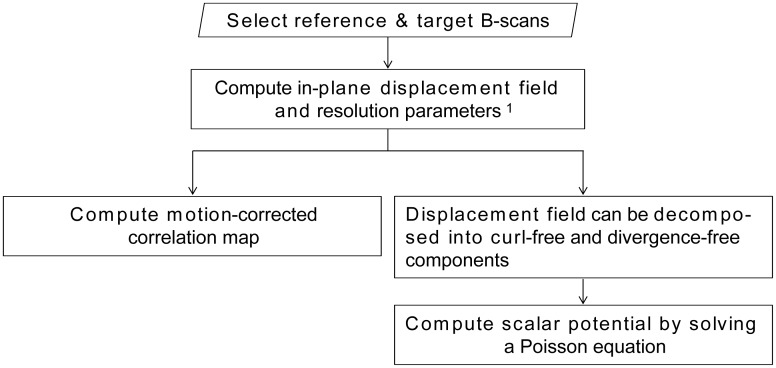
Flowchart of OCT image processing.

The thermal displacement of laser-irradiated retinal tissue was monitored by OCT B-scan images taken before, during, and after the laser irradiation. A two-dimensional (2-D) displacement map was computed between a reference B-scan image that was taken before the laser irradiation and a target B-scan image that was taken after the laser irradiation, using the method previously described [25]. In addition, an OCT correlation map *ρ*_*c*_(*x*, *z*) and the scalar potential of the 2-D displacement *D*(*x*, *z*) were computed, based on an algorithm described in S1 Appendix, where *x* is the in-plane lateral position and *z* is the depth position. The overall processing flow is summarized in Fig 2.

The OCT correlation map is a 2-D map representing a spatially resolved correlation between the reference and target OCT B-scan images. By using this map, we could detect a decorrelated area where alterations of tissue microstructure or large displacement exceeding the measurable range of our displacement measurement method had occurred.

The scalar potential of displacement was computed from the measured in-plane displacement field. A pseudocolor image was generated for easy understanding, where the color hue represents the scalar potential as shown in the color bar, the color saturation represents the magnitude of the in-plane displacement, and the lightness represents OCT intensity. The color hue changes from blue, green to red, with increases of the scalar potential, and therefore, the tissue moves from red regions (high potential) to blue regions (low potential).

### Materials and experimental protocol

Ten *ex vivo* whole porcine eyes, which were obtained from a local abattoir (Daimon Ltd., Ibaraki, Japan), were used. These eyes were enucleated within 12 h after death and kept in a refrigerator, and are investigated within 12 h after enucleation. A custom-made zero diopter contact lens was attached to the cornea to prevent dehydration. We included all eyes which meet the following criteria: first, the retinal fine structure such as nerve fibers and clear boundary between retinal pigment epithelium complex and neural retinal layer were visible in OCT. Second, the dynamic range of signal-to-noise ratio of the OCT image is more than 25 dB. Third, the structure and SNR are stable during the experiment.

To systematically investigate the effects of laser power and exposure times, the laser parameters were changed for each measurement, which were performed on slightly different retinal positions. The laser power (*P*) was varied from a level regarded as subthreshold to that used in standard protocols [6,27]. Specifically, the laser power was configured to 50, 100, 200, 300, or 400 mW. The exposure time (*T*) was configured to 25, 50, 100, or 200 ms. In total, 20 combinations of laser power and exposure time were used in 20 different retinal positions for each eye. All 10 eyes were examined with the same protocol.

During the measurements, a sequence of OCT B-scans was obtained at a frame rate of 80 B-scans/s, with photocoagulation starting 125 ms after the first B-scan image acquisition. The OCT B-scan imaging area was 1.5 mm (lateral) × 1.6 mm (axial), which consisted of 1,024 pixels (lateral) × 512 pixels (axial). To compute tissue displacements, a reference B-scan image was obtained just before laser irradiation, and target B-scan images were obtained at 12.5, 50, 100, 200, and 2000 ms after the start of the laser irradiation. The size of a spatial kernel, which is a parameter of our displacement measurement algorithm [25], were empirically set to 14 pixels (lateral) × 14 pixels (axial). The amount of digital shift, which is another parameter of the displacement measurement algorithm [25], was empirically configured to 2.9 μm for lateral and 3.2 μm for axial.

## Results

### Microstructural alterations of laser-irradiated retinal tissue

The effects of laser irradiation on retinal tissue with different laser parameters were observed in the OCT B-scan images and OCT correlation maps. OCT images were taken with four combinations of laser power and exposure time, which were selected to have the same total laser energy of 10 mJ.

**Fig 3:**
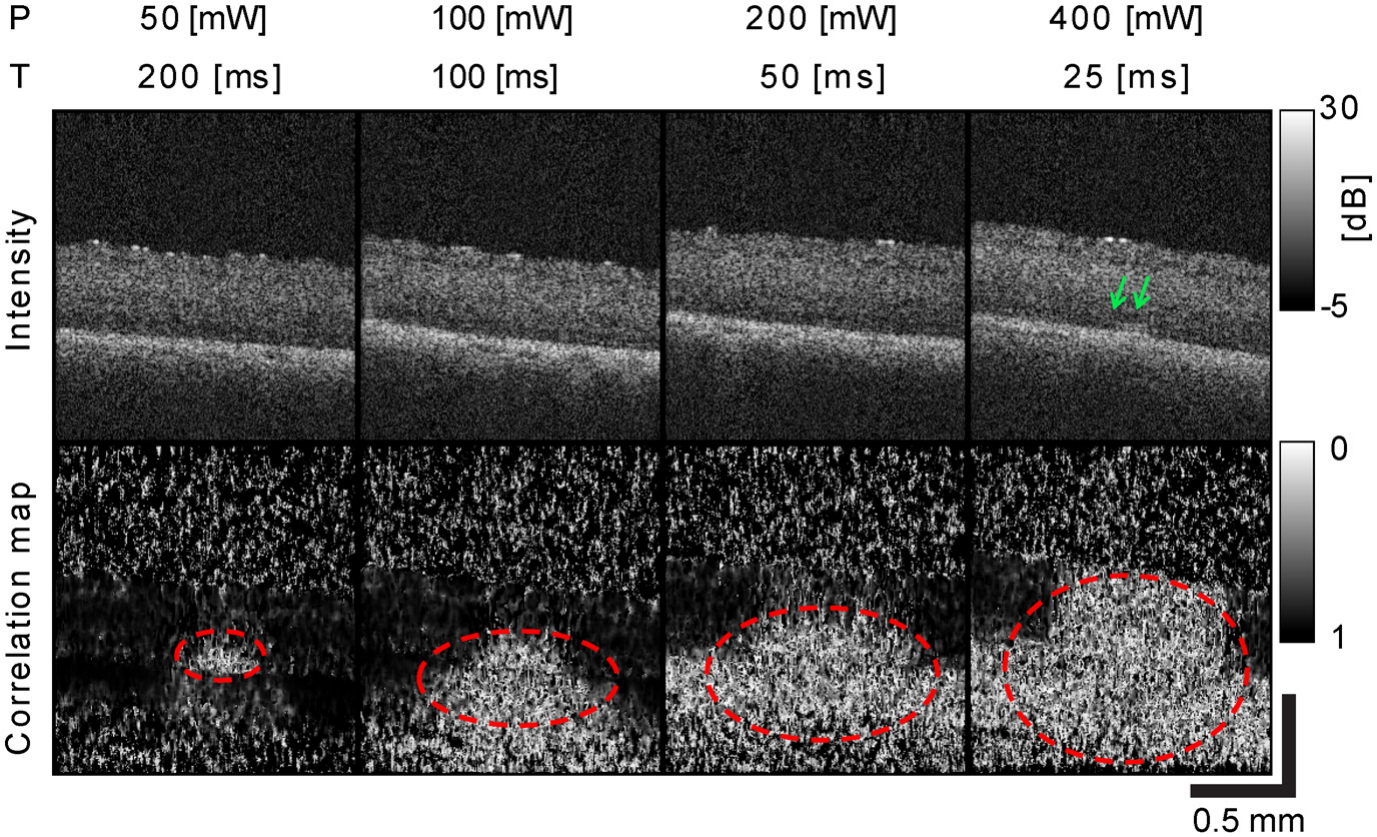
OCT B-scan images and OCT correlation maps with different laser parameters while maintaining the same total energy of 10 mJ. OCT B-scan images (top row) and correlation maps (bottom row) when changing the laser power and duration while maintaining the total energy at 10 mJ.

The top row of Fig 3 shows the OCT B-scan images, with four different laser configurations, taken 2,000 ms after the starting time of laser irradiation. The increase of scattering at the outer retinal layer as indicated by green arrows was visible in no eye for *P* = 50 mW and *T* = 200 ms, no eye for *P* = 100 mW and *T* = 100 ms, in one eye for *P* = 200 mW and *T* = 50 ms, and in eight eyes for *P* = 400 mW and *T* = 25 ms out of 10 eyes. In summary, increased scattering was observed only with irradiation with high power and reduced exposure times.

The bottom row of Fig 3 shows OCT correlation maps that were computed before and after laser irradiation. The time interval between acquisitions of these images was 2,000 ms. Although the same energy of 10 mJ was applied, the decorrelated areas (indicated by red circles) enlarge as laser power increases and exposure times decrease. This increase of decorrelation areas was observed in all 10 eyes. The results suggest that shorter exposure time results in more alteration of tissue microstructure and larger deformations, which is consistent with a previous report based on a histological study [6].

### Kinetic dynamics of tissue during laser irradiation

**Fig 4:**
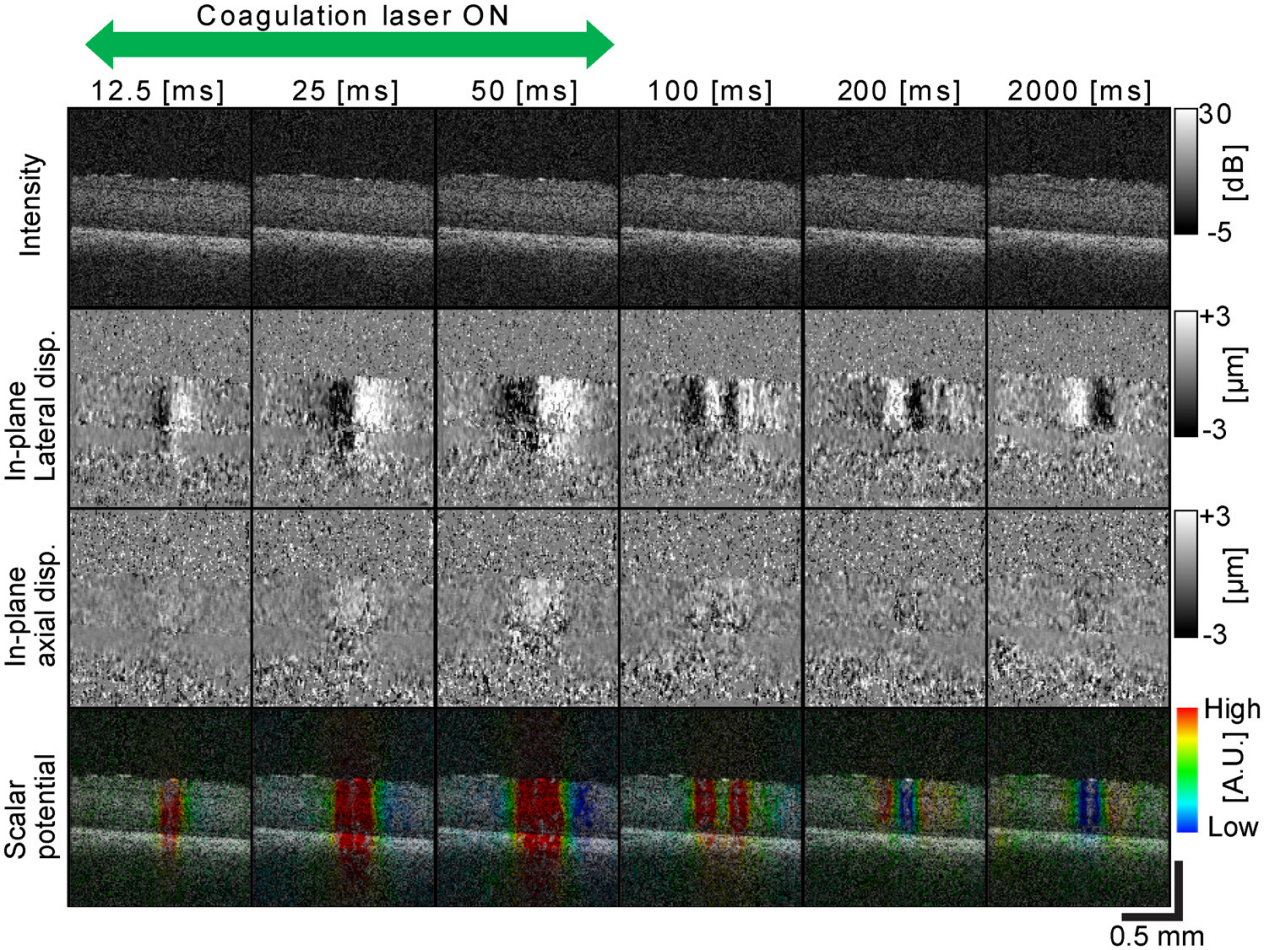
OCT B-scan images, in-plane lateral and axial displacements and scalar potentials at different time points (Class-1). OCT B-scan images (first row), in-plane lateral displacements (second row), in-plane axial displacements (third row), and the color composite images (fourth row) where the color represents the scalar potential at different time points.

**Fig 5:**
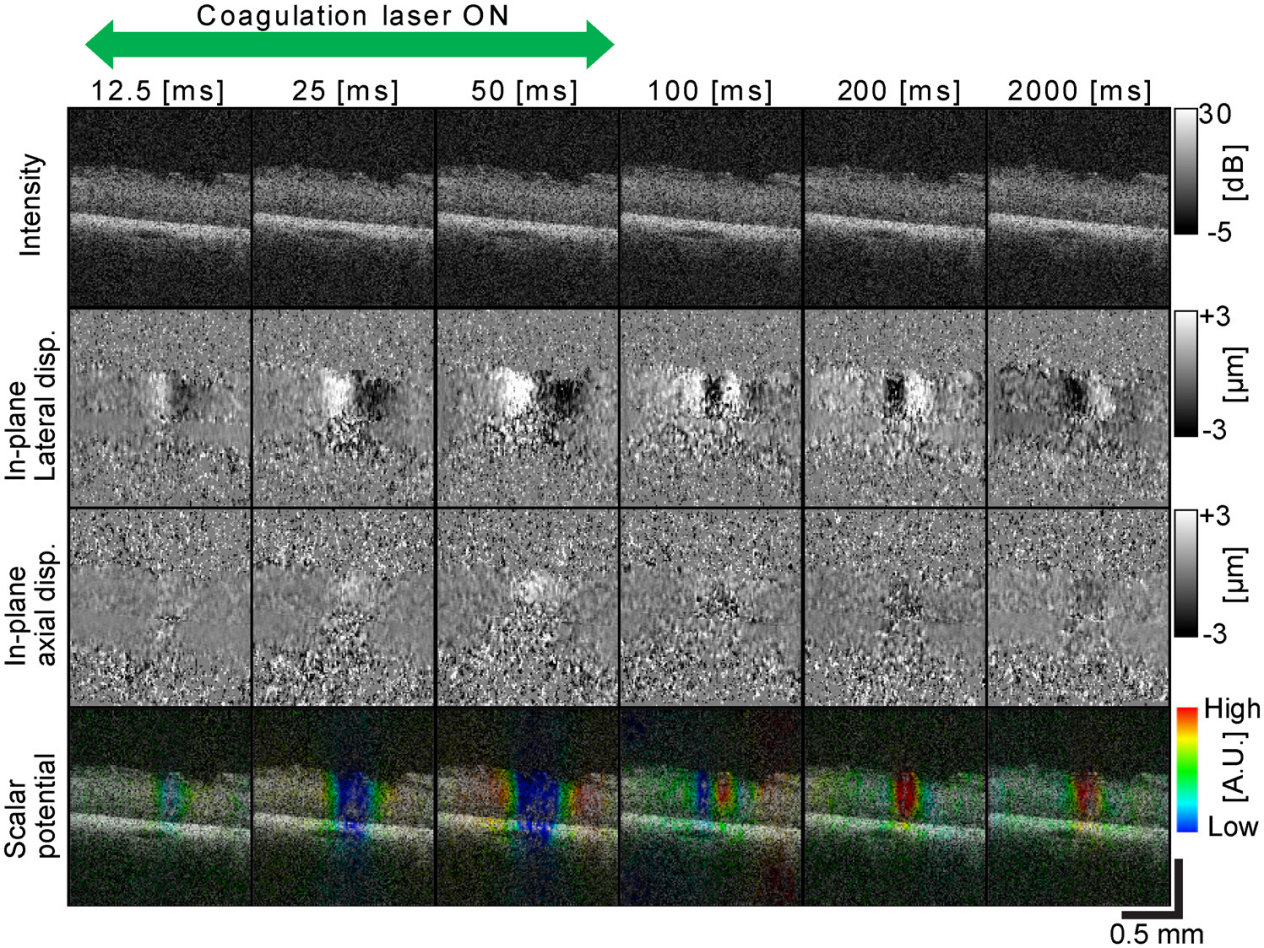
OCT B-scan images, in-plane lateral and axial displacements and scalar potentials at different time points (Class-2). OCT B-scan images (first row), in-plane lateral displacements (second row), in-plane axial displacements (third row), and the color composite images (fourth row) where the color represents the scalar potential at different time points.

The displacement map revealed tissue displacement induced by laser irradiation. Submicrometer displacements were observed at the neural retinal regions with low power laser irradiation (*P* ≤ 200 mW) as shown in Figs 4 and 5 (*P* = 50 mW, *T* = 50 ms). The four rows represent OCT intensity images, in-plane lateral displacement maps, in-plane axial displacement maps, and the scalar potentials of the displacement (from top to bottom), respectively. Each column corresponds to each time point. It is noteworthy that such small displacements could not be visualized in OCT intensity images.

In 67.5% of irradiation spots with high laser power laser (*P* > 200 mW), the tissue displacement was not correctly measured. Using such high power, the amount of tissue displacement exceeds the maximum measurable displacement of our method and/or alteration of tissue microstructure was too severe to be examined by our method. However, such large displacements should be observable by standard OCT images as morphological changes or increases in scattering, so our method is not necessary for these cases.

According to the time course of tissue displacement during and after laser irradiation, laser-induced tissue dynamics was classified into three classes. The first class (Class-1) was characterized by lateral expansion of neural retina during the laser irradiation, followed by lateral constriction after shutting down the irradiation laser as shown in the representative case in Fig 4. As shown in the figure, both lateral and axial displacements were reversed from expansion to constriction after 200 ms. Because tissue displacement would be radially symmetric in the *en face* plane, this change suggests that tissue dynamics in this class involved radial expansion followed by radial constriction. This class of dynamics was observed in 33.3% of irradiation spots among spots with *P ≤* 200 mW.

The second class of dynamics (Class-2) was characterized by the opposite lateral direction of displacement, when compared with the first class. The neural retina was laterally constricted during laser irradiation and then laterally expanded after irradiation. However, the neural retina was axially displaced in the same direction as the first class during and after the laser irradiation. Fig 5 shows a representative case of this class. Lateral constriction changed to lateral expansion at around 100 ms. It would be noteworthy that the images at 2,000 ms time points showed lateral expansion with respect to the reference B-scan (baseline). This class was observed in 37.5% of irradiation spots among the spots with *P ≤* 200 mW.

The cases that could not be categorized into the first two classes were categorized as the third class (Class-3). This class is characterized by the following dynamics. The neural retina was axially displaced in the same direction as the first class. However, in the lateral direction, apparent expansion or constriction was not observed. In an example case, the neural retina showed no apparent lateral displacement. In another example, the neural retina was displaced in the obliquely right upward direction during laser irradiation. Despite this variety of lateral displacements, all cases in this class showed the same dynamics of axial displacement. This class was observed in 29.2% of irradiation spots among the spots with *P ≤* 200 mW.

The number of irradiation spots of each class with several laser power and exposure-time configurations are summarized in Table 1.

**Table 1:** Number of retinal positions classified by dynamics of tissue displacements for each laser parameter.

	50 mW	100 mW	200 mW	All *P*
25 ms	4/3/3 (40.0/30.0/30.0)	3/4/3 (30.0/40.0/30.0)	3/6/1 (30.0/60.0/10.0)	10/13/7 (33.3/43.3/23.3)
50 ms	3/3/4 (30.0/30.0/40.0)	3/4/3 (30.0/40.0/30.0)	3/5/2 (30.0/50.0/20.0)	9/12/9 (30.0/40.0/30.0)
100 ms	3/3/4 (30.0/30.0/40.0)	3/5/2 (30.0/50.0/20.0)	4/5/1 (40.0/50.0/10.0)	10/13/7 (33.3/43.3/23.3)
200 ms	3/2/5 (30.0/20.0/50.0)	4/2/4 (40.0/20.0/40.0)	4/3/3 (40.0/30.0/30.0)	11/7/12 (36.7/23.3/40.0)
All *T*	13/11/16 (32.5/27.5/40)	13/15/12 (32.5/37.5/30)	14/19/7 (35/47.5/17.5)	

The number of retinal positions classified into Class-1, -2, and -3 for 12 combinations of laser power (*P*) and exposure time (*T*). The numbers of the three classes are shown, with a slash to separate Class-1 to -3, from left to right. The numbers in the parentheses correspond to percentages of each class. Only the retinal regions exposed with laser power ≤ 200 mW were presented in this table.

## Discussion

### Benefits of OCT correlation map

The decorrelated region in the OCT correlation map indicates microstructural alteration of the tissue as well as large displacements that exceed the measurable range of our method. This decorrelated region was found even with the lowest power (*P* = 50 mW) and shortest exposure time (*T* = 25 ms). In addition, a larger decorrelation region was observed with higher laser power and shorter exposure time, while the same total energy (10 mJ) was applied, which is consistent with the previous reports [6,7]. Furthermore, we observed that the de-correlated region increased as the laser power increases at each constant irradiation time in all eyes (S1 Fig), and this indicates the temperature dependency of tissue denaturation. Because both large deformations and microstructural alterations are caused by thermal effects such as denaturation, and thermo-elastic and thermo-refractive changes, the OCT correlation map can be used to objectively evaluate thermal tissue changes induced by photocoagulation. This objective evaluation could lead to a further understanding of thermal effects such as histologically and/or ophthalmoscopically defined thermal damage lesion size [6,7] and the correlation with the Arrhenius model of cellular thermal damage [7].

As an objective indicator, the OCT correlation map can lead to high reproducibility of the photocoagulation. Although Lavinky et al., demonstrated highly reproducible photocoagulation with the help of titration protocol, it relies on an ophthalmoscopically determined retinal lesion [28]. The decorrelation region in the OCT correlation map can be an alternative to determine the threshold level, and it is beneficial in particular in *in vivo* measurement [29], because the dosage on the retina is rarely known in particular when we measure patients having severe retinal disorder and cataract. It is also worth mentioning that the quantitative characterization of tissue scattering may further improve the objective evaluation of thermal damage. For example, the light attenuation coefficient analysis [30] at irradiated retinal region can be determined without knowing the opacity of intermediate ocular media.

In addition to the small changes detected by the OCT correlation map at the time of photocoagulation therapy, the retinal structure deforms slowly after the laser irradiation, over a longer time scale to form scars [15,31]. These healing and scar formation processes are still not well known. Therefore, in practice, a follow-up examination is required to evaluate the therapeutic effects. Standard OCT is a practical postoperative examination tool for this purpose, because chronic and large structural deformations can be observed in standard OCT B-scan images [15].

The OCT correlation map would also be useful as a postoperative examination tool, because of its sensitivity to small motions that include ocular flow, as similar to OCT-based angiography [32]. Intervention in ocular blood flow is one of the important roles of photocoagulation. It regresses the neovascularization, prevents the formation of edema, and reduces the release of vascular endothelial growth factor. Therefore, the correlation map can provide additional use as a follow-up and complement to standard OCT.

### Kinetic dynamics of neural retina

According to time-course displacement measurement results, the classification of tissue dynamics and laser parameters did not show a straight-forward relationship. It may be because the kinetic dynamics of lateral displacement during laser irradiation varies among eyes, even with the same laser configuration. This variation in dynamics can be explained by inhomogeneity and the inter-eye-variation of laser absorption and mechanical properties of retinal tissue [33,34]. On the other hand, the variation in thermal properties do not seem to explain this observation. It is because the thermal properties of biological tissues are mainly dominated by water content, which is generally regarded as homogeneous [35,36] and would not highly vary among eyes. In other words, the variation in dynamics may be sensitive to the spatially localized thermal changes and mechanical properties of each specific eye and retinal location. These distinctive features are essential to understand and describe the thermal change at the neural retina in response to an increase in temperature. Therefore, the classification results could lead to a further understanding of thermal damage on the neural retina, which is important in particular at the subthreshold level.

It should also be added that the same dynamics of axial displacement was found among three classes, which is consistent with the previous report by Müller et al [17]. As described, it can be expected that there is a linear relationship between temperature and axial displacements, and its slope depends on certain threshold irradiance. In our displacement measurements, the irradiance was from 924 [W/cm^2^] to 3,696 [W/cm^2^]. Although it does not cover their entire range of previous studies, it is reasonable to believe our method has similar ability to phase sensitive measurement [17] for the investigation of the kinetic dynamics associated with axial displacement. In addition, the method allows us to measure the kinetic dynamics of lateral displacement, which may lead to further understanding of the relationship between the displacements and temperature.

### Limitations

These observations were owing to our developed algorithm which enabled in-plane and out-of-plane tissue displacements using correlation coefficients of OCT [25], and our method has an advantage of measuring more complicated dynamics than previous work [17]. However, it has several limitations as follows: first, the method can visualize but not quantify the deformation. Second, in *in vivo* measurement [29], the method is sensitive to involuntary eye motion, and therefore requires the additional image registration algorithm.

Although the results have shown that our method can be used as an objective indicator of thermal damage on the retina, there are some limitations in this study as follows. At first, the light dosage on the retina was less considered. This is because our approach was derived from an assumption that the dosage on the retina is unknown. Namely, our method is designed to give an objective evaluation without knowing the exact parameters on the retina, which is particularly important for future clinical measurement *in vivo*. Although this assumption makes direct comparison difficult, our results substantiated the significant variability between eyes and retinal locations even with the same laser diameter. Second, thermal lesion size is not defined histologically nor ophthalmoscopically. Although the results of OCT correlation map are consistent with previous histological studies, it is not directly comparable with other studies [6,7]. Third, the post-mortem change, such as changes in blood concentration, temperature, deformation, is not taken into account. In vivo experiment would be required for further understanding of the photocoagulation process. However, even with enucleated eyes, our results showed evidences to provide an objective indicator of thermal damage on the retina, in particular, the OCT correlation map showed consistent results with histological study. This is an important step to understand the results obtained from in vivo measurement [29].

To the best of our knowledge, this is the first study showing 2-D cross-sectional dynamic tissue displacements during photocoagulation at a submicrometer scale. Because this method provides objective displacement information, it would provide further understanding of thermal tissue changes occurred by laser irradiation.

## Supporting Information

S1 Appendix Motion-corrected correlation map and scalar potential. (PDF)

S1 Fig OCT correlation maps with different laser parameters. OCT correlation maps for all combinations of laser power (column) and exposure time (row).
(PDF)
