# Supplementary material for: Investigation of Thermal Effects of Photocoagulation on Retinal Tissue Using Fine-Motion-Sensitive Dynamic Optical Coherence Tomography
Source: PLoS One. 2016 Jun 6;11(6):e0156761. doi: 10.1371/journal.pone.0156761 (PMC4894600; doi:10.1371/journal.pone.0156761)
Supplement: S1 Appendix — (PDF) [file pone.0156761.s001.pdf]

S1 Appendix. Motion-corrected correlation map and scalar potential.

We employed a post-processing method to compute the OCT correlation map, the scalar potential of displacement from the measured tissue displacements, and the resolution parameters that were obtained as previously described [1]. In this supplementary section, the details of the algorithms of the OCT correlation map and the scalar potential are described.

Motion-corrected correlation map

The correlation map was obtained by computing the local correlation coefficients between a reference OCT B-scan and a target B-scan. The correlation coefficient between OCT B-scans decreases by both a rigid body displacement and a nonrigid deformation, such as an alteration of microstructure and random motion of the scatterers. So the OCT correlation map visualize these two.

In our algorithm, bulk in-plane subpixel displacements induced by photocoagulation were removed by dividing the raw measured correlation coefficient $\rho_{measured}(x,z)$ by an expected correlation coefficient computed from the in-plane motion as

$$\rho_c(x,z) = \rho_{measured}(x,z) \exp\left[\frac{\Delta x^2(x,z)}{w_x^2(x,z)} + \frac{\Delta z^2(x,z)}{w_z^2(x,z)}\right], \qquad (1)$$

where $\rho_c(x,z)$ is the bulk displacement corrected correlation coefficient. The exponential term is the inverse of the expected correlation coefficient, which represents a reduction in the correlation coefficient occurring only by displacement. $\Delta x(x,z)$ and $\Delta z(x,z)$ are spatially resolved lateral and axial

displacements, respectively, obtained from the displacement map. $w_x(x,z)$ and $w_z(x,z)$ are lateral and axial resolution parameters obtained from our displacement measurement algorithm [1].

Note that the bulk displacement correction is accurate only if the displacements are rigid. Nonrigid deformations, alterations of tissue microstructure, and large displacements exceeding the maximum measurable range reduce the accuracy of the correction.

Scalar potential

Scalar potential was computed from the measured in-plane displacement field $\vec{a}(x,z) = \left(\Delta x(x,z), \Delta z(x,z)\right)$.

On the basis of Helmholtz's theorem, a general displacement field $\vec{A}(x,z)$ can be decomposed into an irrotational (curl-free) component, which is associated with a scalar potential, and an incompressive (divergence-free) component, which is associated with a vector potential, as

$$\vec{A}(x,z) = \nabla D(x,z) + \nabla \times \vec{R}(x,z), \qquad (2)$$

where $D(x,z)$ is a scalar potential and $\vec{R}(x,z)$ is a vector potential. Then, the divergence of the above equation yields the following Poisson equation,

$$\Delta D(x,z) = \nabla \cdot \vec{A}(x,z). \qquad (3)$$

By assuming a boundary condition that no displacements existed at the perimeter of the field, the scalar potential of the irrotational displacement component was obtained as [2,3]

$$D(x,z) = -\mathrm{FT}^{-1}\left[\frac{\mathrm{FT}\left[\nabla \cdot \vec{A}(x,z)\right]}{k_x^2 + k_z^2}\right], \quad (4)$$

where $\mathrm{FT}[\ ]$ represents a 2-D Fourier transform from the (x,z)-domain to the spatial frequency domain, i.e., (k_x, k_z) – domain. $\mathrm{FT}^{-1}[\ ]$ represents the inverse Fourier transform.

In our implementation, $\vec{A}(x,z)$ was obtained by applying the median filter to the measured displacement field $\vec{a}(x,z)$ with a kernel size of 25 × 15 pixels, and the discrete Fourier transform was used to perform the Fourier transform.

References

1. Kurokawa K, Makita S, Hong Y-J, Yasuno Y. In-plane and out-of-plane tissue micro-displacement measurement by correlation coefficients of optical coherence tomography. Optics Letters. 2015 May 1;40(9):2153.

2. Stam J. Stable Fluids. In: Proceedings of the 26th Annual Conference on Computer Graphics and Interactive Techniques [Internet]. New York, NY, USA: ACM Press/Addison-Wesley Publishing Co.; 1999 [cited 2015 Jan 28]. p. 121–8. Available from: http://dx.doi.org/10.1145/311535.311548

3. Hinkle J, Fletcher PT, Wang B, Salter B, Joshi S. 4D MAP Image Reconstruction Incorporating Organ Motion. In: Prince JL, Pham DL, Myers KJ, editors. Information Processing in Medical Imaging [Internet]. Springer Berlin Heidelberg; 2009 [cited 2015 Jan 26]. p. 676–87. Available from: http://link.springer.com/chapter/10.1007/978-3-642-02498-6_56
