# Supplementary figures and images for: Investigation of Thermal Effects of Photocoagulation on Retinal Tissue Using Fine-Motion-Sensitive Dynamic Optical Coherence Tomography
Source: PLoS One. 2016 Jun 6;11(6):e0156761. doi: 10.1371/journal.pone.0156761 (PMC4894600; doi:10.1371/journal.pone.0156761)

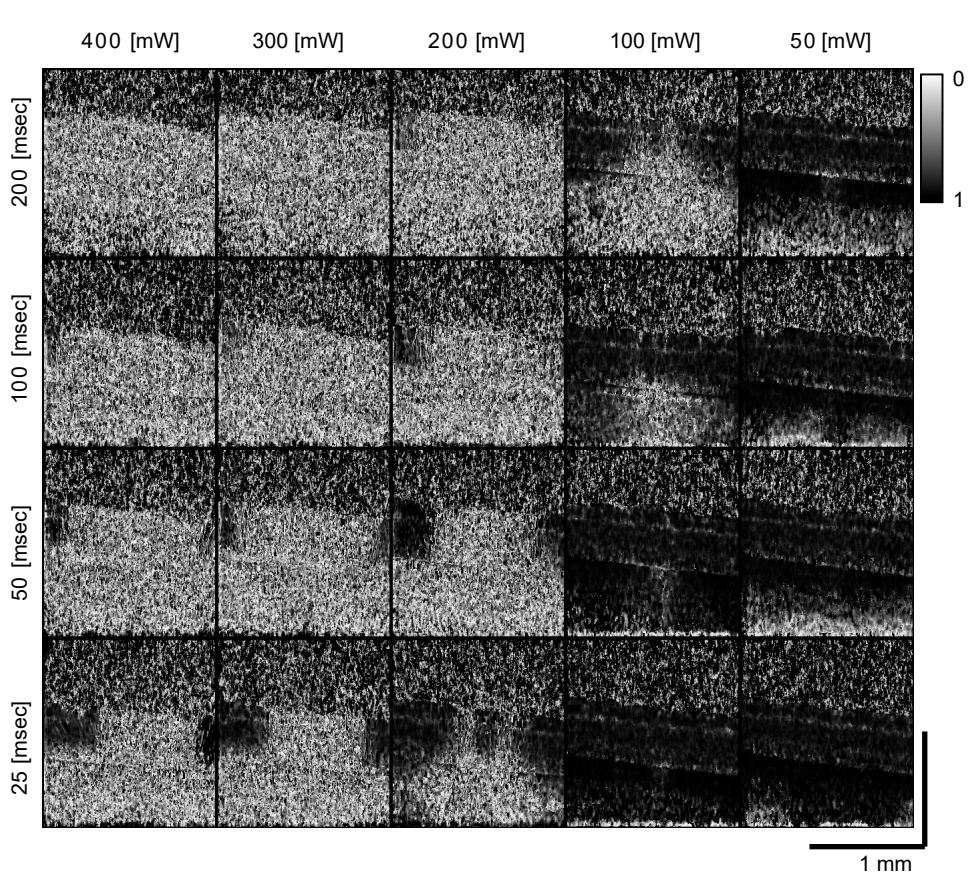

Supplement: S1 Fig — OCT correlation maps for all combinations of laser power (column) and exposure time (row). (PDF) [file pone.0156761.s002.pdf]
